# Does empathy decline in the clinical phase of medical education? A study of students at Leicester medical school

**DOI:** 10.1016/j.pecinn.2024.100316

**Published:** 2024-07-02

**Authors:** Leila Keshtkar, Andy Ward, Rachel Winter, Char Leung, Jeremy Howick

**Affiliations:** a Stoneygate Centre for Empathic Healthcare, Leicester Medical School, University of Leicester, George Davies Centre, Lancaster Rd, Leicester LE1 7HA, UK; b Leicester Medical School, University of Leicester, George Davies Centre, Department of Population Health Sciences, Lancaster Rd, Leicester LE1 7HA, UK

**Keywords:** Empathy, Medical school, Education

## Abstract

**Objective:**

To examine whether medical student empathy changes throughout the five years of a UK medical school.

**Methods:**

Students completed an online version of the Jefferson Scale of Empathy (JSE-S) during the 2022–2023 academic year. Comparisons of empathy scores were made using analysis of variance (ANOVA), and independent *t*-tests.

**Results:**

Empathy scores varied across different years of medical school (*P* ≤ 0.001), with a small drop in empathy between the pre-clinical and clinical phases of medical school (Mean difference = 1.82, *P* = 0.025). Male students scored lower than female students and there was no statistically significant difference between the mean empathy score and speciality interest.

**Conclusions:**

Students' empathy appeared declined slightly as they progressed through medical school. As a crucial component of good clinical care, interventions in medical education to enhance empathy should be prioritised.

**Innovation:**

This is the first time following the COVID-19 pandemic that medical student empathy was measured across all five years of a medical school. Unlike many previous related studies, we identified the point at which empathy appears to decline, providing guidance for educators who can target empathy enhancing interventions where they are most needed.

## 1 Introduction

Empathy is considered the cornerstone of the clinician-patient relationship and a key element of healthcare practitioner professionalism [1]. Clinician empathy can improve diagnoses and clinical outcomes [[2], [3], [4]]. Increased empathy is also linked to greater job satisfaction and lower burnout in healthcare practitioners [5,6]. Not all research on empathy is positive, with one systematic review suggesting that affective empathy could increase burnout [7]. Research has also shown that empathy is a skill that can be taught [8].

There are many definitions of empathy, and the concept of empathy overlaps with other concepts, especially compassion [9]. Additionally, empathy is widely recognised as being multidimensional, interpersonal, modulated by context, and includes cultivating empathic concern [10]. A recent systematic review has found that most definitions of empathy include understanding, feeling, and self–other differentiation [9].

Despite the potential for empathy to improve patient care, most [[11], [12], [13], [14]], but not all [[15], [16], [17]] studies that have measured it have found that medical student empathy declines as they progress throughout medical school. Erosion of empathy in medical school could be attributed to several factors. A stressful working environment, time pressures, poor role modelling, focus on biomedical models in teaching, tick-box empathy training, limited experience and lack of emotional “bandwidth” can impact students' ability to overcome the hurdles that stand in the way of developing and maintaining empathy [18]. Different authors have reported that modifications to medical school curricula can lead to improvements rather than a decline in empathy [15,18]. To design effective interventions that enhance medical student empathy, it is important to examine whether empathy does, in fact, decline within that setting, and if so, when the decline is most serious.

The purpose of this study was to compare empathy levels among students from different years in medical school using a validated self-reported measure of medical student empathy [19].

## 2 Methods

A cross-sectional study was conducted with students from all five years of medical education at Leicester Medical School.

### 2.1 Participants

Students undertaking the undergraduate medicine degree during the 2022–2023 academic year were invited to take part.

### 2.2 Study survey

The Jefferson Scale of Empathy student-version (JSE-S) was used. The JSE-S is a scale developed by Hojat et al. for measuring clinical empathy in the context of health profession education and patient care [19]. The scale contains 20 items, which are answered on a seven-point Likert scale (1 = strongly disagree, 7 = strongly agree). To control for a tendency to passively and consistently endorse “agree” (or “disagree”) responses to the test questions, 10 items are positively worded (directly scored) and 10 items are negatively worded (reverse scored). The possible range of scores is 20–140, with higher scores representing higher levels of empathy.

Participants were also asked to choose their future career interests from a list of medical options (as one the JSE questionnaire): “Community-based,” “Surgical,” “Medical,” “Mental health,” and “Unknown or undecided” (Appendix A). This is because some specialities may require a higher degree of empathic engagement due to the frequency of encounters, the nature of consultations, and the provision of continuous care. These different specialities may attract students with different abilities and aptitudes for empathy, and it is important to explore whether this influences empathy scores.

### 2.3 Procedures

The online JSE-S was administered to students during year-specific protected teaching time. Participant information and consent forms were integrated into the online surveys. Completion rates were based on the numbers in the whole year group, rather than on those present at the time of the surveys. Apart from the voluntary option to enter emails for receiving feedback, no personal identification information was solicited or collected. All individual data were anonymised.

### 2.4 Statistical analyses

We used Analysis of variance (ANOVA) to examine changes in the JSE-S scores. One-way ANOVA was computed to assess differences in total scores related to the year of medical school and speciality preference. In this analysis, the JSE-S score served as the dependent variable, the study groups by year in medical school, and speciality interest were the independent variables. Significant effects were followed by post hoc paired comparison tests using the Tukey Honestly Significant Difference (HSD) test. In an additional analysis, empathy scores between students in different phases of medical school were combined and compared by using an independent *t*-test. An independent t-test was also used to test the significance of the differences in pairwise comparisons according to gender and year of medical school training. A *P*-value of less than <0.05 was considered to indicate statistical significance. All computations were performed using SPSS version 28 for Windows (IBM, New York, USA).

### 2.5 Ethics

The project and associated documents were approved by the University of Leicester Research Ethics Committee (Ethical approval number: 37516-rw205-ls: medicine).

## 3 Results

At the time of the surveys, 1534 students were undertaking their degree at Leicester Medical School. This included 312, 302, 299, 302, and 319 students in the first to fifth years, respectively. In total, 890 (58.0%) of all students completed the survey: 258 (82.7%) first-year students, 223 (73.8%) second-year students, 115 (50.2%) third-year students, 175 (57.9%) fourth-year students, and 119 (37.3%) fifth-year students (Table 1).

**Table 1:** Characteristics of survey participants.

Year	n/N (%)	% Female	Medical speciality group interest: n/N(%)				
			Community-based	Surgical	Medical	Mental health	Undecided
1	258/312 (82.7%)	62.8	15/258 (0.06)	41/258 (0.16)	74/258 (0.29)	14/258 (0.05)	114/258 (0.44)
2	223/302 (73.8%)	62.8	27/223 (0.12)	34/223 (0.15)	49/223 (0.22)	6/223 (0.03)	107/223 (0.48)
3	115/229 (50.2%)	73.0	14/115 (0.12)	26/115 (0.23)	39/115 (0.34)	4/115 (0.03)	32/115 (0.28)
4	175/302 (57.9%)	67.4	26/175 (0.15)	31/175 (0.18)	66/175 (0.38)	8/175 (0.04)	44/175 (0.25)
5	119/319 (37.3%)	74.8	24/119 (0.20)	25/119 (0.21)	43/119 (0.36)	2/119 (0.02)	25/119 (0.21)

Descriptive statistics for the empathy scores in each year of medical school for the total participants are presented in Table 2.

**Table 2:** Empathy scores for students in each year of medical school.

Year	Number of participants	Empathy score Mean (Standard Deviation)
1	258	115.02 (9.41)
2	223	112.00 (11.54)
3	115	113.21 (13.43)
4	175	110.42 (11.55)
5	119	113.62 (12.47)

The first year had the highest empathy score, with the lowest score in year four.

### 3.1 Empathy changes and year of medical school

We found a statistically significant difference in mean empathy scores by year of medical school (*P* < 0.001). A post hoc ANOVA pairwise mean comparisons test was also conducted, which indicated statistically significant differences in mean empathy scores between students in years one and two (*P* = 0.004), years one and four (*P* < 0.001), years three and four (*P* = 0.042), and years four and five (*P* = 0.018).

The means and standard deviations of combined empathy scores between students in the preclinical phase (years one and two) and the clinical phase (years three, four and five) are summarised in Appendix B. The independent *t*-test did not reveal a statistically significant difference in combined empathy scores between students in the preclinical phase compared with the clinical phase (*P* = 0.054). However, taking into account that students in the first semester of year three have not yet engaged fully with the clinical years of medicine, and following previous studies suggesting that medical student empathy appears to change in the third year [17,18], we compared combined JSE-S score from years one, two, and three with those of years four and five (Appendix C). This analysis revealed a small statistically significant drop in empathy score (Mean difference = 1.82, 95% Confidence Interval: 0.22, 3.43, *P* = 0.025).

### 3.2 Empathy changes and gender

**Fig. 1:**
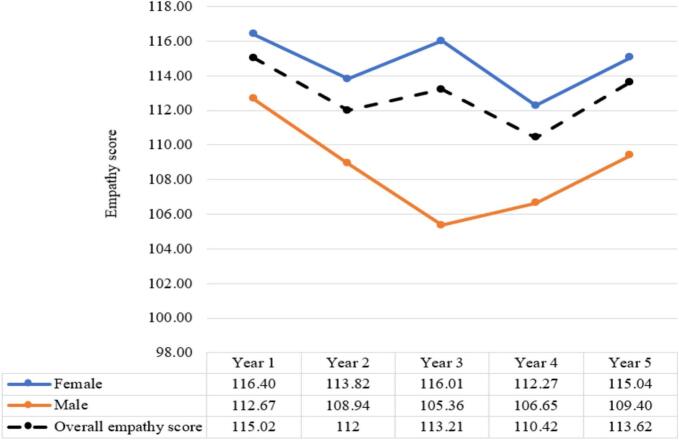
Comparison of scores on the JSE-S for male, female and the overall empathy score during medical school.

The means and standard deviations of combined empathy scores based on gender for all years are provided in Appendix D. Results indicated that male respondents had slightly lower empathy scores than females in all medical school years (see Fig. 1), and the average difference was statistically significant (Mean difference = 5.32, 95% Confidence Interval: 3.75, 6.89, *P* < 0.001).

### 3.3 Empathy changes and speciality of interest

There was no statistically significant difference in JSE scores by speciality interest (*P* = 0.40).

## 4 Discussion and conclusion

### 4.1 Summary of results

This study showed that empathy appears to decline by a very small amount as students' progress through medical school, and male students had marginally lower empathy scores than female students. Indicated speciality of interest did not appear to affect empathy scores.

### 4.2 Comparison with other evidence

Previous systematic reviews found mixed evidence regarding whether medical student empathy goes down as they progress throughout medical school [20,21]. Some studies report little or no change [22,23], and many studies found a decline in empathy [13,21]. In 2020, a systematic review including 30 studies, 14 reported a decline in medical student empathy levels, four reported increasing empathy levels, six found no statistically significant difference in empathy scores and the remaining studies were ambiguous [21]. A scoping review of 20 studies of medical student empathy from 2017 [20], suggests that there is little evidence to support a generalised trend in changes in student empathy throughout medical school. By indicating a small decline in empathy, our findings dovetail with many other research findings in the field [13,21,22] and added that the decline in empathy appears to occur as students transition to the clinical phases of their education.

Consistent with previous studies, our results found that female medical students score more highly than males [24,25]. Whilst other studies, however, have found differences in empathy scores in students with different speciality interests [[26], [27], [28]], ours did not.

Compared with a systematic review of empathy levels among medical schools from before the pandemic [21], our results were broadly similar. However, our study would have to be replicated to confirm whether the pandemic did not cause a change in empathy levels. Future research should investigate whether the pandemic caused a change in medical student empathy and why the pandemic may have had such an effect.

### 4.3 Innovation

This is the first time since the COVID-19 pandemic that medical student empathy was measured in all five years of study at a large UK medical school. We were also able to identify the point at which empathy appeared to decline, namely as students transition from the pre-clinical to clinical phases of their education. Providing the details about the timing of empathy decline indicates to educators where they can target empathy interventions to reverse any decline.

### 4.4 Study limitations and recommendations for future research

There are some potential limitations to consider. The tool used to measure empathy, the JSE-S is self-reported, potentially influencing participants to provide a more positive response than is reflected by their actual behaviour [20,22,29]. It also measures medical students' orientation to empathy and does not measure actual behaviour [11]; some students might believe they are very empathic, yet do not demonstrate empathic behaviours [16]. Further research may examine the specific relation of the JSE-S in real medical practice by complementary tools such as supervisor ratings or peer reviews and also consider the assessment of student empathy from the perspective of real or simulated patients. Mitigating this limitation, our aim was to measure the change in empathy, and such a change may not be sensitive to the limitations of the scale.

Additionally, it is important to note that first-year students (82.7%) had a higher rate of participation in the survey compared to fifth-year students (37.3%). This could have distorted our findings.

Furthermore, our study is cross-sectional and not a longitudinal follow-up, so we were unable to observe changes in empathy in the same cohort of students as they progressed through medical school. A longitudinal study could improve our understanding of contributing factors in the variation of empathy and address the role of medical education systems and medical curricula that can prevent the erosion of empathy. Future research should address this limitation.

Moreover, there are confounders such as age, student demographics and teaching methods that are not controlled for in this study. Relatedly, this study only shows whether there are differences in empathy scores but does not identify factors that might explain the differences. Future research is now warranted to identify, develop, and deliver interventions that reverse the decline in medical school empathy [30].

Furthermore, while the evidence overwhelmingly points to the need for additional empathy [[1], [2], [3], [4], [5], [6]] it could be that there is an optimal level of empathy, beyond which there is no additional benefit, or indeed a harm. For example, a decline in empathy might be beneficial if it leads to a reduction in burnout. Future research should attempt to determine whether there is an optimal level of empathy and if so, what it is.

Finally, there are different types and components of empathy (e.g., cognitive, affective, and behavioural) and JSE focuses mainly on measuring clinical empathy [31] and another scale could be used to capture different aspects of empathy and provide a different result.

## 5 Conclusion

Empathy is an important skill that can impact patient and future practitioner health. By showing that empathy may decline, and when this decline is likely to occur, this study provides a rationale for introducing targeted empathy enhancing interventions to medical students.

## Source of funding

This study was funded by the Stoneygate Trust.

## Data sharing

None.

## CRediT authorship contribution statement

**Leila Keshtkar:** Writing – original draft, Methodology, Formal analysis, Data curation. **Andy Ward:** Writing – review & editing, Data curation, Conceptualization. **Rachel Winter:** Writing – review & editing, Data curation, Conceptualization. **Char Leung:** Writing – review & editing. **Jeremy Howick:** Writing – review & editing, Supervision, Methodology, Funding acquisition, Conceptualization.

## Declaration of competing interest

The authors declare that they have no known competing financial interests or personal relationships that could have appeared to influence the work reported in this paper.

## Figures and Tables

**Table:** 

Medical speciality group interest	Group
General practice	Community-based
Public health	
General surgery	Surgical
Neurosurgery	
Obstetrics and gynaecology	
Otolaryngology	
Orthopaedic surgery	
Plastic surgery	
Urology	
Anaesthetics	Medical
Dermatology	
Emergency medicine	
General medicine	
Neurology	
Ophthalmology	
Pathology	
Paediatrics	
Radiology	
Psychiatry	Mental health

**Table:** 

Medical school year	Number of participants	Empathy score mean (SD)	Std. Error mean
Years 1 and 2 (pre-clinical)	481	113.61 (10.57)	0.4821
Years 3, 4 and 5 (clinical)	409	112.12 (12.47)	0.6168

**Table:** 

Medical school year	Number of participants	Empathy score mean (SD)	Std. Error mean
Years 1, 2 and 3	595	113.54 (11.18)	0.4583
Years 4 and 5	295	111.71 (12.05)	0.7019

**Table:** 

Gender	Number of participants	Empathy score mean (SD)	Std. Error mean
Female	593	114.71 (9.86)	0.4052
Male	297	109.38 (13.55)	0.7864
